# Wide Response Range Photoelectrochemical UV Detector Based on Anodized TiO_2_-Nanotubes@Ti@quartz Structure

**DOI:** 10.3390/nano14050439

**Published:** 2024-02-28

**Authors:** Youqing Wang, Miaomiao Zhang, Wenxuan Wu, Ze Wang, Minghui Liu, Tiantian Yang, Renqianzhuoma

**Affiliations:** 1 Research Center for Semiconductor Materials and Devices, Shaanxi University of Science and Technology, Xi’an 710021, China; 2 School of Mechatronic Engineering, Xi’an Technological University, Xi’an 710021, China

**Keywords:** UV detectors, photoelectrochemical, TiO_2_ nanotubes, anodization, response range

## Abstract

Conventional sandwich structure photoelectrochemical UV detectors cannot detect UV light below 300 nm due to UV filtering problems. In this work, we propose to place the electron collector inside the active material, thus avoiding the effect of electrodes on light absorption. We obtained a TiO_2_-nanotubes@Ti@quartz photoanode structure by precise treatment of a commercial Ti mesh by anodic oxidation. The structure can absorb any light in the near-UV band and has superior stability to other metal electrodes. The final encapsulated photoelectrochemical UV detectors exhibit good switching characteristics with a response time below 100 ms. The mechanism of the oxidation conditions on the photovoltaic performance of the device was investigated by the electrochemical impedance method, and we obtained the optimal synthesis conditions. Response tests under continuous spectroscopy confirm that the response range of the device is extended from 300–400 nm to 240–400 nm. This idea of a built-in collector is an effective way to extend the response range of a photoelectrochemical detector.

## 1. Introduction

UV light refers to light with wavelengths ranging from 10 nm to 400 nm, which has numerous beneficial uses and unfavorable effects [1,2]. Therefore, it is important to quantitatively detect UV light. The UV detector is a device that converts light signals into electrical signals and is designed to detect UV light [3]. In recent years, UV detectors have been widely used in fields such as light curing, environmental monitoring, biomedicine, military, fire fighting, cultural relics protection, etc. [4,5,6,7,8,9]. Compared with traditional photoconductive UV detectors, a new type of photoelectrochemical (PEC) UV detector that has recently emerged has attracted much attention due to its advantages such as a simple preparation process, easy availability of raw materials, and no need for additional external voltage [10].

The traditional PEC UV detector consists of three parts: photoanode, electrolyte and counter electrode [11]. The photoanode is mainly composed of a transparent conductive substrate (such as FTO/ITO) and a semiconductor nanostructured thin film [12]. As the core part of the entire device, the role of the photoanode is to absorb UV light, generate electron–hole pairs, and conduct photogenerated electrons [10]. Ni et al. prepared a core–shell-structured self-powered UV photodetector with MgO-modified TiO_2_ nanowires (NWs) using FTO glass as a conductive electrode, which has high responsiveness and high switching ratio [13]. However, due to the filtering effect of FTO/ITO thin films on UV light, traditional PEC UV detectors cannot detect UV light below 300 nm. To address the above issues and broaden the response range, Wei et al. reported a newly designed PEC UV detector that used a special stainless-steel-mesh-based electrode through which UV light can directly reach the active material. The detector showed good photoelectric conversion ability and considerable switching performance, even under very weak irradiation [14]. Furthermore, Wang et al. prepared a novel PEC device with Ag NWs as conductive electrodes, quartz glass as the substrate, and TiO_2_ nanoparticles as photoactive layers, and the response range of the UV detector was extended [15]. However, all of the above devices are complex to fabricate and have unstable performance. Both stainless steel and Ag metal are unstable under I^−^/I_3_^−^ electrolyte systems, which seriously affects the service life of the devices [16]. In addition, the active material attached to the electrode surface also falls off very easily during long-term operation.

As a wide band gap semiconductor, TiO_2_ has received widespread attention due to its good photochemical properties, stability, easy availability of raw materials, and nontoxicity. Various methods for preparing nanostructured TiO_2_ active layers have been reported. Compared with simple TiO_2_ nanoparticles, TiO_2_ nanotubes (NTs) have a larger specific surface area, faster electron transmission speed and lower electron recombination efficiency [17,18,19]. The electrochemical anodizing method as a common method for preparing TiO_2_-NTs generally uses pure Ti materials as the anode, and graphite or Pt sheets as the cathode [20]. Electrochemical oxidation can prepare TiO_2_-NTs with three-dimensional (3D) structures on different Ti substrates. Due to the ability of TiO_2_-NTs to absorb reflected and refracted light, the photon loss caused by scattering effects in the electrolyte can be minimized [21,22,23,24]. Currently, the TiO_2_-NTs/(FTO/ITO) structures have been applied in dye-sensitized solar cells (DSSC) and photocatalysis [25,26,27]. As far as we know, there are no reports of applying anodizing TiO_2_-NTs to the PEC UV detector.

In this work, we used the anodization method to generate the TiO_2_-NTs network structure on the Ti mesh in situ. Under precise control, the unoxidized Ti wire inside can serve as a conductive electrode to conduct photogenerated electrons to the external circuit. Finally, quartz glass with high transmittance throughout the entire UV region was used as the substrate to form a TiO_2_-NTs@Ti@quartz (abbreviated as TOTQ) type PEC-UV detector. This particular collector-built PEC UV detector successfully solves the problem of UV utilization of conventional structures and broadens the response range of the device from 300–400 nm to 240–400 nm. The in situ one-step preparation process improves the stability of the photoanode, while the all-Ti material system can be adapted to various electrolytes.

## 2. Materials and Methods

**Figure 1:**
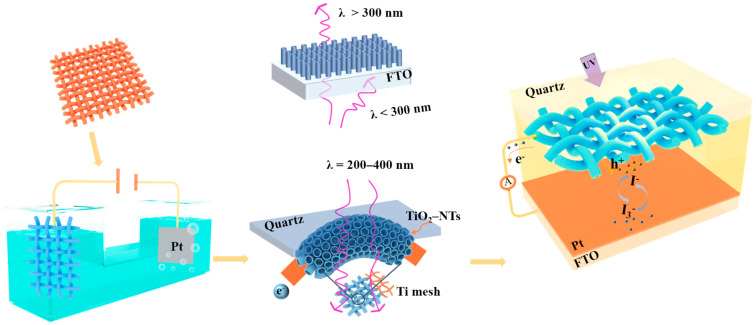
The preparation process and working principle of TOTQ PEC UV detector. The cleaned Ti mesh is anodized to form NTs, which are then attached to quartz glass to form a photoanode. PEC UV detector can be formed by packing the photoanode with the counter electrode and injecting electrolyte in the middle.

All the chemicals used in the experiment were analytical grade without further purification. The preparation process of the PEC-UV detector based on TOTQ is shown in Figure 1.

### 2.1. Preparation of Photoanodes

The raw material of Ti mesh used was sponge Ti with a purity of 99.5%. Firstly, the Ti mesh was cut into small square pieces of 8 × 14 mm^2^, which were then cleaned with acetone, absolute ethanol, and deionized water for 5 min, respectively. The traditional two-electrode system was used for anodic oxidation with Ti mesh as the working electrode and a Pt sheet with purity above 99.99% as the counter electrode. The distance between the two electrodes was about 2.5 cm. Anodization was carried out under the drive of a direct-current (DC) power supply (120 V, 3 A). The electrolyte consisted of 0.25 wt% NH_4_F + 0.75 vol% H_2_O glycol solution [28]. The oxidation was divided into two groups: control the oxidation voltage and oxidation time, respectively. The first group was oxidized at 15, 30, 45, 60, 75 and 90 V for 60 min, and the second group was oxidized for 30, 60, 90, 120, 150 and 180 min at 60 V. After anodization, the samples were rinsed with deionized water and then ultrasonically cleaned for 30 s. The purpose of ultrasonic cleaning was to remove the organic impurities on the oxide surface. After treatment, the samples were annealed in a muffle furnace at 550 °C for 3 h [29]. 

### 2.2. Encapsulation of PEC UV Detectors

The prepared TiO_2_-NTs/Ti mesh was pasted on the quartz glass; then, the counter electrode was packaged and the distance between the photoanode and the counter electrode was controlled to be about 100 μm. Finally, a drop of electrolyte was injected into the gap. The counter electrode was prepared by dropping the H_2_PtCl_6_ solution onto the FTO glass and annealing at 400 °C for 30 min. The iodine-based electrolyte contained 1.12 g of 1.3-dimethylimidazole iodide, 0.038 g of iodine, 0.06 g of guanidine thiocyanate, 0.34 g of tert-butyl pyridine, 0.0335 g of lithium iodide, 4.25 mL of acetonitrile, and 0.75 mL of butyl cyanide. See previous works for more details [30,31].

### 2.3. Characterization

TiO_2_-NTs were prepared with a DC power supply. The microstructure of TiO_2_-NTs/Ti mesh was observed with a field emission scanning electron microscope (FESEM, Hitachi S-4800). UV transmission performance was tested with a UV-vis photometer. The crystal structure was tested with an X-ray diffractometer (XRD). The detection performance of PEC UV detectors was tested with the electrochemistry workstation.

## 3. Results and Discussion

### 3.1. Characteristics of TiO_2_-NTs@Ti Mesh Photoanode

**Figure 2:**
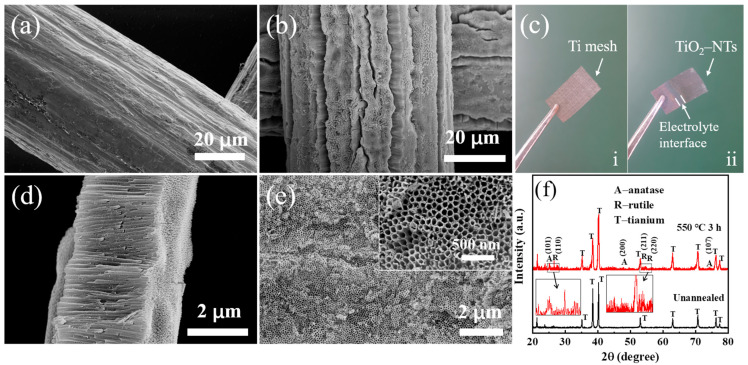
SEM morphology of (**a**) Ti mesh and (**b**) anodized Ti mesh. (**c**) Physical image of Ti mesh before and after anodizing, i (before oxidation), ii (after oxidation). (**d**) Characterization of the length of TiO_2_-NTs. (**e**) Morphological characterization of TiO_2_-NTs. (**f**) XRD spectrum of TiO_2_-NTs@Ti mesh.

The morphology and XRD patterns of the Ti mesh before and after anodization are shown in Figure 2. The surface of the Ti mesh is smooth with no cracking before anodization (Figure 2a). As shown in Figure 2b, the TiO_2_-NTs prepared by anodic oxidation on Ti mesh finally show cracks, which are mainly based on the difference perimeter between the bottom and the top of the connection point, as well as the van der Waals force and capillary force during the drying process [26]. The cracks between NTs are conducive to the diffusion of the electrolyte, but if the cracks are too deep, they will lead to a recombination reaction between the Ti core in the middle and the electrolyte directly. Figure 2c shows the digital images of the Ti mesh before (i) and after (ii) anodization. The color of the Ti mesh changes from silver gray to dark gray, and the reaction is intensified due to the rapid transfer of electrons at the electrolyte interface, leading to faster formation of NTs and higher transmittance of the Ti mesh. We peeled the TiO_2_-NTs formed by anodizing at 60 V for 60 min from the Ti substrate; as shown in Figure 2d,e, the average length of the TiO_2_-NTs is 2.8 μm, the inner diameter is 46.82 nm, and the wall thickness is 18.11 nm. High-temperature annealing is an important means to transform TiO_2_ from the amorphous phase to the anatase and rutile phases, and a high content of the anatase phase of TiO_2_ is conducive to electronic transmission. The XRD pattern after annealing at 550 °C is shown in Figure 2f. After high-temperature treatment for 3 h, the TiO_2_-NTs are transformed into the anatase phase. Since the anatase phase of TiO_2_ starts to transform into the rutile phase at around 450 °C, the diffraction peaks of the rutile phase can also be seen in the plots.

**Figure 3:**
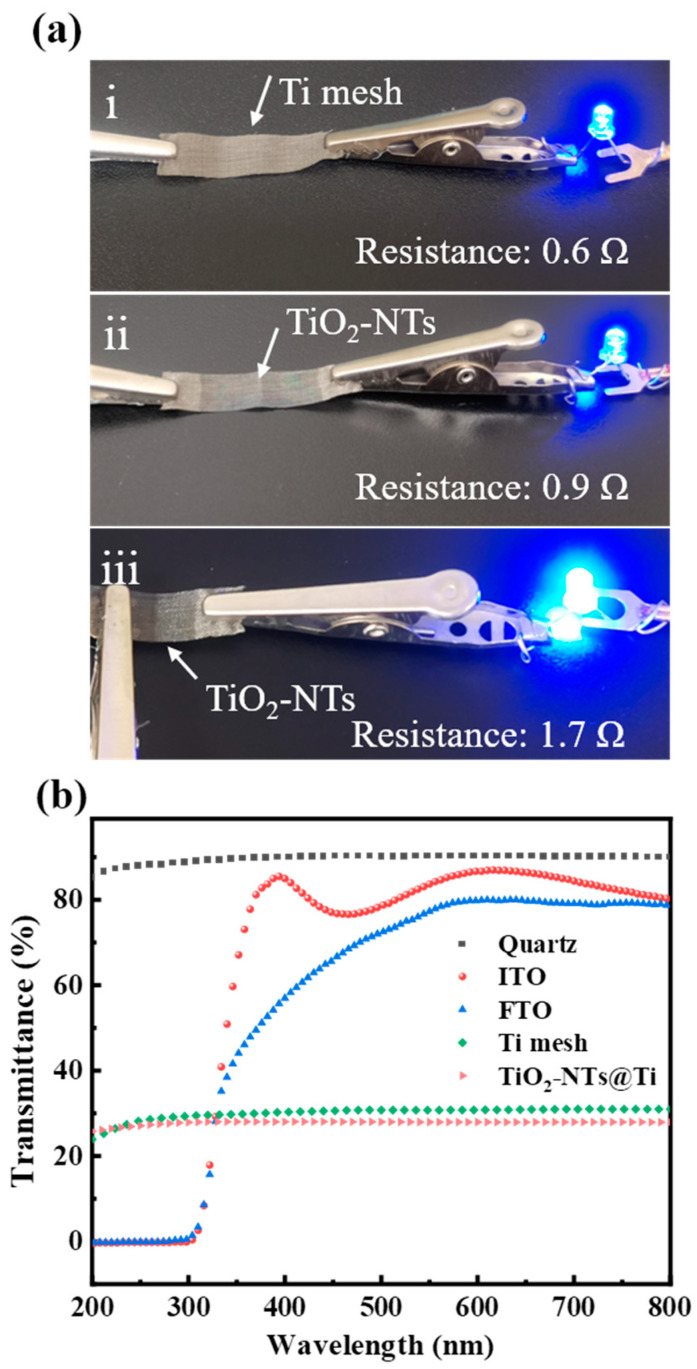
(**a**) Changes in resistance of Ti mesh at both ends before and after anodizing (90 V, 90 min), before anodizing (i), after anodizing (ii), between TiO_2_-NTs and Ti mesh (iii). (**b**) Transmission spectrum of FTO, ITO, quartz, Ti mesh, and TiO_2_-NTs@Ti mesh.

It is important to ensure that the Ti core in the center has good electrical conductivity after oxidation. As illustrated in Figure 3a, the resistance of the Ti mesh before anodization is 0.6 Ω, and the conductivity remains stable after 90 min of anodization at 90 V, with a resistance of 0.9 Ω. The resistance between the Ti-conducting channels at both ends and the NT surface is also low at 1.7 Ω. This provides a strong guarantee for the transport of photogenerated carriers. The TiO_2_-NTs@Ti structure obtained by anodizing on the surface of the Ti mesh naturally has the function of a photoanode for the PEC UV detector. Therefore, when the packaging devices, only a high transmittance substrate needs to be added. The curves in Figure 3b show the transmittance of five different substrates. Due to the filtering problem of FTO/ITO to UV light, traditional devices cannot respond below 300 nm. In contrast, quartz glass has a high transmittance of 90% in the entire near-UV and visible regions, which can be used as an ideal substrate material for PEC UV devices. Therefore, in the subsequent study, we chose quartz glass as the substrate to form the TOTQ-structured PEC UV detector. On the other hand, the Ti mesh has a low transmittance of about 30%, indicating that most of the light sources can be effectively utilized, and there is no significant difference in transmittance between the UV and visible regions. The slight decrease in the transmittance of TiO_2_-NTs@Ti after anodizing indicates that more light is being utilized, which is due to the light scattering induced by the NT structure that enhances the light absorption.

### 3.2. Performance of PEC UV Detectors

**Figure 4:**
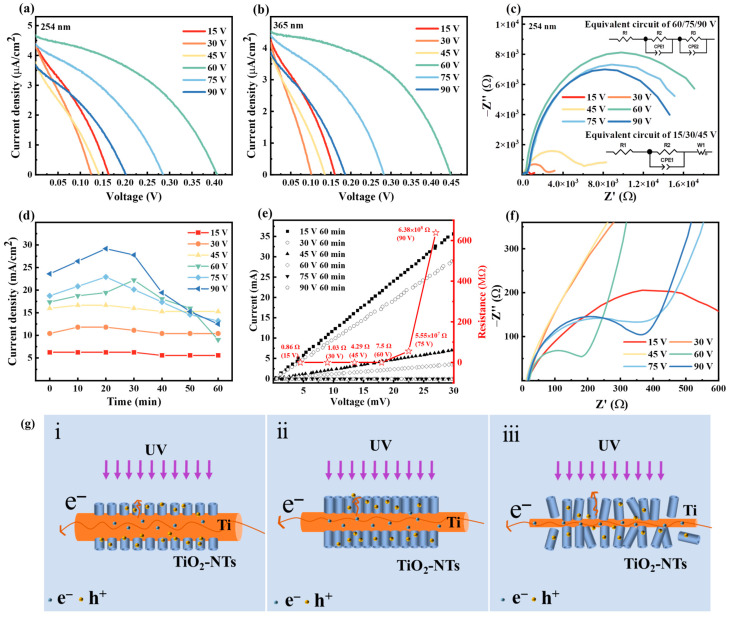
(**a**,**b**) J–V curves of the PEC UV detector based on different oxidation voltages for 60 min at 254 nm and 365 nm, respectively. (**c**) Electrochemical impedance spectroscopy (tested at 254 nm), the illustration shows the equivalent circuit diagram. (**d**) Time-dependent curve of anodic oxidation current density. (**e**) Changes in resistance of Ti mesh under different oxidation voltages. (**f**) Partial enlarged view of Figure 4c. (**g**) Schematic diagram of Ti mesh oxidation,low voltage (i), optimum voltage (ii), high voltage (iii).

The volt ampere characteristic test is an important characterization to reflect the photovoltaic conversion capability of PEC UV detectors [32]. Figure 4a,b show the J–V curves of devices prepared under different oxidation voltage conditions, where Figure 4a refers to the tests at 254 nm (300 μW/cm^2^), and Figure 4b refers to the tests at 365 nm (180 μW/cm^2^).

As the oxidation voltage increases, the open circuit voltage (V_oc_) and short circuit current density (J_sc_) of the device show a trend of first increasing and then decreasing, and the best performance comes from the device prepared at 60 V. The V_oc_ and J_sc_ of the device reach 0.45 V and 4.53 μA/cm^2^ at 365 nm, respectively. When the voltage is too low, it is difficult to produce an effective anodic oxidation reaction, and very little TiO_2_-NT is formed, which subsequently affects light absorption and charge exchange at the interface. When the voltage is too high, the rapid response causes the destruction of the nanostructure, while the internal Ti core is overconsumed, introducing a large charge transfer impedance. SEM images of samples obtained at different voltages are shown in Figure S1. It can be seen that when the voltage is low, it is difficult to form a tubular structure, and only a TiO_2_ nanofilm is formed instead.

We further investigated the mechanism of the reaction voltage on the performance by impedance spectroscopy. The Nyquist map is used to characterize electron transfer between interfaces [33]. Usually, the electrochemical impedance spectrum consists of high-frequency semicircles and low-frequency oblique straight lines or semicircles. The spectrum composed of high-frequency semicircles and low-frequency oblique lines represents the charge transfer and diffusion processes. When the spectrum is composed of high-frequency small semicircles and low-frequency large semicircles, the small semicircle represents the impedance (R_ce_) of the interface between the counter electrode and electrolyte, and the large semicircle represents the impedance (R_ct_) of the electron recombination process at the interface between the photoanode and electrolyte [34,35]. By fitting the Nyquist diagram with an equivalent circuit (illustrated in Figure 4c), the series resistance (R_s_) and charge transfer resistance (R_ce_/R_ct_) can be estimated.

The intersection of the Nyquist curve and the real part represents the series impedance (R_s_), which is mainly derived from the resistance of the electrolyte [34]. Due to the use of iodine-based electrolytes in the same amount, the solution resistance of each device is maintained at around 15 Ω. The curves in Figure 4c show that the Nyquist spectrum consists of high-frequency semicircles and low-frequency oblique lines when the voltage is 15 V, 30 V, and 45 V. As the voltage increases, the radius of the semicircle increases. This may be due to the increase in voltage leading to an increase in the roughness of the generated TiO_2_ film, and the charge transfer resistance increases. The consistent slopes of the low-frequency oblique line indicate that the diffusion rate of electrons in the electrolyte is the same. When the voltage is 60 V, 75 V, and 90 V, the Nyquist curve consists of two semicircles, where the diameter of the low-frequency small semicircle increases as the voltage increases, while the diameter of the high-frequency large semicircle decreases as the voltage increases. The difference in morphology and structure between the NTs and the nanofilms leads to a change in the electronic conduction of the devices during operation, which changes the Nyquist pattern.

The relationship between the current density and the time of oxidation reaction is shown in Figure 4d. As the voltage increases, the current density of the oxidation reaction increases and the oxidation reaction becomes more intense until 20 min of oxidation, when the current density reaches its maximum value. At the beginning of the anodic oxidation reaction, the Ti mesh loses electrons and reacts with water in the electrolyte to form a TiO_2_ oxide layer, resulting in an increase in current density. When the reaction lasts for about half an hour, the TiO_2_ oxide layer is gradually corroded by F^-^ in the electrolyte to form a porous structure, and the current density decreases with time. When the electrochemical oxidation rate and chemical dissolution rate tend to be equal, the current density no longer changes, and the prepared TiO_2_-NTs tend to stabilize and no longer change. In this work, when the oxidation potential was set to 60 V, it was found through monitoring that the current density did not change after 90 min of oxidation (Figure S2). The I–V curves and resistance of TiO_2_-NTs@Ti photoanode structures prepared under different oxidation voltages are shown in Figure 4e. The linear test results indicate that no Schottky barriers are present in the structure. The resistance of Ti mesh increases with the increase in oxidation voltage. When the voltage is greater than 60 V, the generated TiO_2_-NTs have very poor contact with the Ti substrate due to the intense reaction. After high-temperature calcination, TiO_2_-NTs will separate from the substrate, resulting in poor conductivity. This result further confirms that 60 V is the optimal preparation voltage.

**Figure 5:**
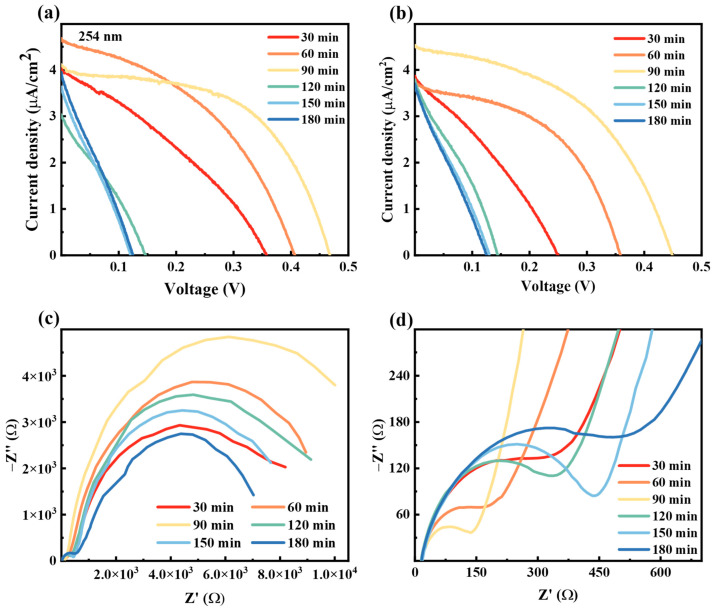
(**a**,**b**) J–V performance test of samples obtained at different preparation times (preparation voltage control to 60 V). (**c**,**d**) Electrochemical impedance spectroscopy (tested at 254 nm).

Figure 5a,b display the photovoltaic characteristic of the device measured by controlling the oxidation time under the oxidation voltage of 60 V. As the oxidation time changes, the V_oc_ and J_sc_ of the device both show a trend of increasing first and then decreasing later, reaching the optimal value at an oxidation time of 90 min. Figure 5c,d show the Nyquist spectra of devices corresponding to different oxidation times. The results indicate that the sample has the lowest electron transfer impedance and the highest electron recombination impedance under the optimum synthesis parameter.

As the device is illuminated, TiO_2_ absorbs UV photons to produce photogenerated electrons, which diffuse along the TiO_2_-NTs to the Ti core for transport. When the oxidation time is short or the oxidation voltage is low, the NTs are not formed or are relatively short and sparse, which does not ensure that most of the UV light is absorbed (as shown in Figure 4g(i)). When the oxidation time is longer or the oxidation voltage is higher, the NTs are prone to fracture and peeling, resulting in the electrolyte directly contacting the Ti core, which in turn leads to a severe recombination reaction between the electrons in Ti and the ions in the oxidation state (as shown in Figure 4g(iii)). In addition, besides the depth of the NT array, the oxidation voltage has an important effect on the morphology and structure of the NTs. Therefore, it is important to optimally control the oxidation time and oxidation voltage considering the light absorption, carrier transport and recombination.

**Figure 6:**
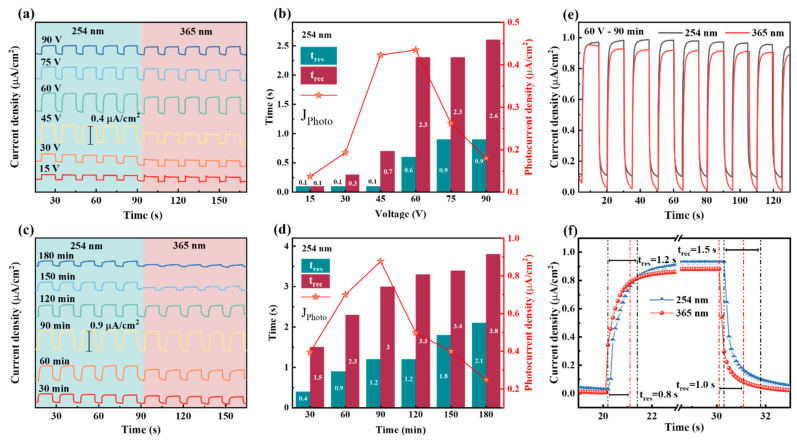
(**a**,**c**) Photocurrent response corresponding to different oxidation voltages and oxidation times. (**b**,**d**) Measurement of response speed under different preparation conditions (t_res_ indicates the response time and t_rec_ indicates the recovery time). (**e**) The switching performance of the UV detectors. The switching period is 15 s. (**f**) Enlarged time response curves.

In order to study the sensitivity of TOTQ PEC UV detectors, we irradiated the devices with UV light in the form of rectangular pulses, with a switching time interval of 15 s. The response speed of the device is mainly affected by carrier diffusion and interface reaction rate [32]. The results show that the detector can operate normally at 0 V bias. As shown in Figure 6a,c, the devices exhibit good switching response at both 365 nm (UVA band) and 254 nm (UVC band). The relative response current under different conditions is consistent with the volt-ampere characteristic test results, and the optimal synthesis conditions are 60 V and 90 min. Under the best conditions, the switching response curve is nearly rectangular, and the current responds very quickly with the light source, and the response current is close to 1 μA/cm^2^. The time sensitivity of the device can be obtained by enlarging the response side and recovery side of the switching curve; as shown in Figure 6f, the typical response time and recovery time are both about 1 s, which is much faster than the photoconductive device.

The time response curve of the device consists of a rising edge of the photo-current response and a falling edge of the dark reaction, and the rising curve is mainly affected by the rapid excitation and transmission of electrons under UV light, while the falling curve is mainly affected by the charge diffusion after stopping illumination. Figure 6b,d show the statistical data of response time (t_res_) and recovery time (t_rec_) of devices under different conditions. As the oxidation voltage increases, both the t_res_ and t_rec_ of the device increase, and the t_rec_ of the same device is longer than the t_res_. This may be due to the fact that with the increase in oxidation voltage, the TiO_2_ active layer becomes thicker, increasing the diffusion distance of photogenerated carriers, and thus extending the response time and recovery time. It should be noticed that the t_res_ and t_rec_ of the device remain between 0.1 s and 0.2 s when the voltage is less than 60 V. This is because the TiO_2_ nanofilm generated at a lower voltage has better contact with the Ti substrate, resulting in faster electron transfer. However, when the voltage is 60 V or above, more TiO_2_-NT structures are generated, and after high-temperature treatment, the separation of the NT from the Ti substrate hinders electron conduction. The length of preparation time also has a similar effect on the response speed.

### 3.3. Extension of the Detection Range

Conventional PEC UV detectors use transparent conductive oxide (TCO) as electrodes to form a sandwich-like structure. During operation, the UV light needs to penetrate the TCO substrate (FTO or ITO) to reach the active layer; however, the TCO substrate has a severe filtering effect on the UV light, which affects the responsiveness of the device and leads to a narrow response range (300–400 nm). Most of the current research work focuses on the synthesis and modulation of the photoanode active material, neglecting the important parameter of response range. The TiO_2_-NTs@Ti core–shell structure obtained by in situ anodic oxidation in this work successfully solved the above problem. (1) The electron collector of the TOTQ structure is located inside the active material, and the UV light of any wavelength can reach the TiO_2_ through the encapsulated quartz without obstacles, which greatly improves the utilization of the light source of the device and thus broadens the response range. (2) The structure with the built-in collector can shorten the diffusion distance of photogenerated electrons, which is conducive to the enhancement of the photo response. (3) The mechanical and chemical stability of the one-step synthesized all-Ti-based structure is superior to the work reported in the literature, for example, the use of stainless steel mesh loaded with the active substance as the electrode, and the use of Ag NWs as the UV-transparent electrode [14]. In addition, TiO_2_-NTs have a large specific surface area due to their unique tubular structure, which can reduce the recombination rate of photogenerated electron–hole pairs and provide directional channels for electrons, enabling the device to have a fast response.

**Figure 7:**
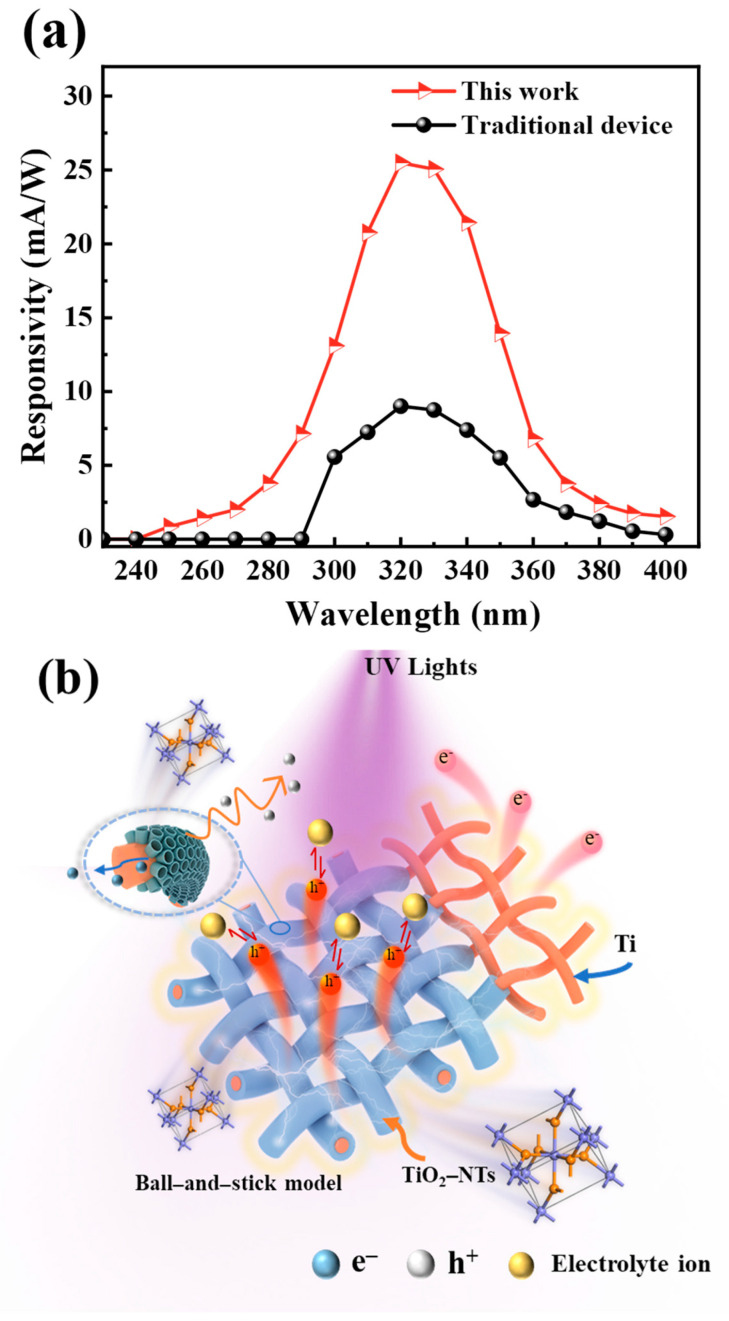
(**a**) Comparison of response spectra between traditional FTO/P_25_ device and the TOTQ PEC UV detector. (**b**) Schematic diagram of generation and transfer of photogenerated electron–hole pairs in TOTQ system.

We tested the response spectra of the devices using continuous calibrated monochromatic light. As shown in Figure 7a, the PEC UV detector based on the TOTQ structure responds in the 240–400 nm range, with the response peak (25.49 mA/W) appearing at 320 nm, which is 69% higher than previously reported devices based on Ag NW electrodes. As a comparison sample, the device based on FTO and TiO_2_ P25 active layer only detects UV light above 300 nm. Figure 7b shows the UV light is irradiated onto the active layer of TiO_2_-NTs through quartz glass and generates electron–hole pairs. The electrons are transmitted along the Ti mesh to the outer circuit, and the holes diffuse to the electrolyte interface to undergo an oxidation reaction with iodine ions.

## 4. Conclusions

In order to solve the problem of the narrow response range of conventional PEC UV detectors, the idea of placing the collector inside the active layer is proposed in this work. A TiO_2_-NTs@Ti core–shell structure was prepared by the anodizing method using a commercially available Ti mesh, together with the use of quartz with high UV transmittance as the substrate to obtain a special TOTQ photoanode structure. The TOTQ photoanode allows UV light of any wavelength to reach the active material directly while shortening the diffusion distance of photogenerated electrons. The one-step-synthesized all-Ti-based structure has better mechanical and chemical stability in iodine-based electrolytes. We precisely controlled the synthesis conditions and determined the optimal oxidation parameters as 60 V and 90 min, and the mechanism of the material structure’s influence on the device performance is clarified by the analysis of electrochemical impedance spectra. The UV test results show good switching performance and high sensitivity, with a response time of 10^2^–10^3^ ms. Compared with conventional devices, the TOTQ PEC UV detector extends the detection range from 300–400 nm to 240–400 nm.

## Data Availability

The data presented in this study are available on request.

## Supplementary Materials

The following supporting information can be downloaded at: https://www.mdpi.com/article/10.3390/nano14050439/s1, Figure S1: SEM morphology of Ti mesh after oxidation at 15/30/45/60/75/90 V for 60 min; Figure S2: Curve of oxidation current density over time.
