# Supplementary material for: Wide Response Range Photoelectrochemical UV Detector Based on Anodized TiO2-Nanotubes@Ti@quartz Structure
Source: Nanomaterials (Basel). 2024 Feb 28;14(5):439. doi: 10.3390/nano14050439 (PMC10934836; doi:10.3390/nano14050439)
Supplement: Supplementary file 1 [file nanomaterials-14-00439-s001.zip › nanomaterials-2867939-supplementary.pdf]

# Supplementary Materials

# Wide Response Range Photoelectrochemical UV Detector Based on Anodized $TiO_2$-Nanotubes@Ti@quartz Structure

**Youqing Wang [1,*], Miaomiao Zhang [1], Wenxuan Wu [1], Ze Wang [1], Minghui Liu [1,2], Tiantian Yang [1]
and Renqianzhuoma [1]**

[1] Research Center for Semiconductor Materials and Devices, Shaanxi University of Science and Technology, Xi'an 710021, China; zhangmiaomiao0504@gmail.com (M.Z.); wuwenxu4n@163.com (W.W.); wze101214@gmail.com (Z.W.); liuminghui_edu@163.com (M.L.); yttmail547@163.com (T.Y.); renqianzhuoma@gmail.com (R.Z.)

[2] School of Mechatronic Engineering, Xi'an Technological University, Xi'an 710021, China

* Correspondence: wangyouqing@sust.edu.cn

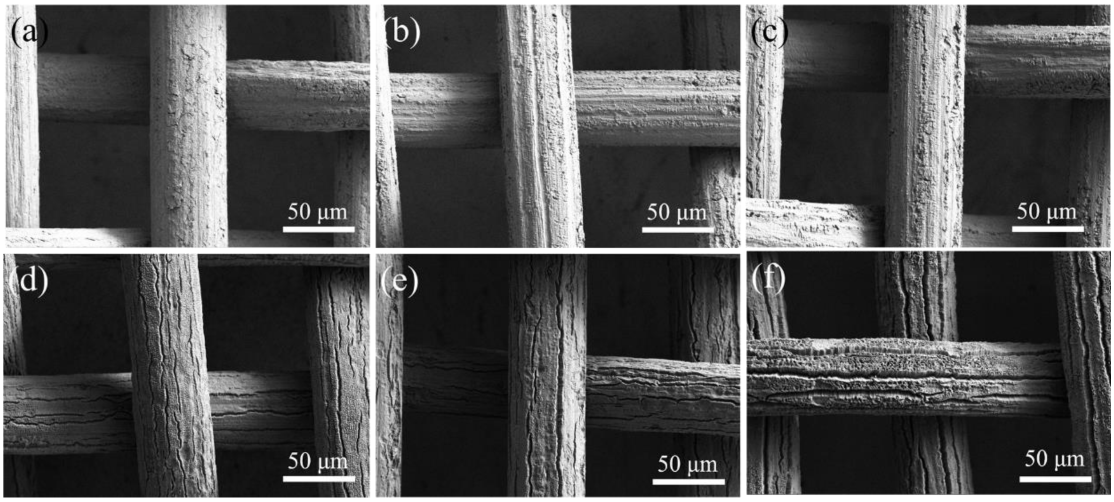


**Figure S1.** SEM morphology of Ti mesh after oxidation at 15/30/45/60/75/90 V for 60 min.

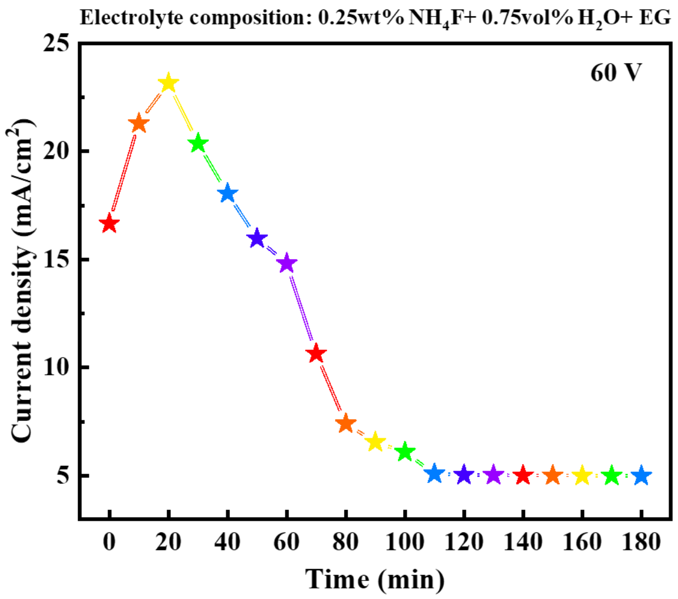


**Figure S2.** Curve of oxidation current density over time.
